# FD-YOLO-Skin: frequency-domain enhanced YOLO for single-class skin lesion detection

**DOI:** 10.3389/fmed.2026.1871697

**Published:** 2026-08-13

**Authors:** Shuang Zhang, Xianyu Zeng

**Affiliations:** 1 Hubei University of Chinese Medicine, Wuhan, China; 2 Department of Dermatology, Traditional Chinese and Western Medicine Hospital of Wuhan, Tongji Medical College, Huazhong University of Science and Technology, Wuhan, Hubei, China

**Keywords:** FD-YOLO-Skin, frequency-domain contrastive learning, frequency-domain multi-scale feature fusion, medical image analysis, skin disease detection, YOLOv8

## Abstract

**Introduction:**

Automatic detection of skin lesions in dermoscopic images remains challenging due to large intra-class variation, low-contrast boundaries, and severe foreground-background imbalance.

**Methods:**

We propose FD-YOLO-Skin, a frequency-domain enhanced YOLOv8 framework for single-class *micronucleus* lesion detection. FD-YOLO-Skin introduces (i) a Frequency-Domain Multi-Scale Feature Fusion (FMSFF) module in the neck to fuse low-frequency shape cues with high-frequency texture details via FFT/IFFT-based multi-branch spectral processing, and (ii) a Frequency-Domain Contrastive Learning (FDCL) module on the backbone that applies spectral augmentations and a contrastive objective to improve feature robustness under complex backgrounds.

**Results:**

On the ISIC-Style Micronucleus Lesion Detection Benchmark (ISIC-MLD; 10,015 de-identified dermoscopic images), FD-YOLO-Skin achieves an mAP@0.5 of 0.990 ± 0.003 (95% CI: [0.986, 0.994]) and an mAP@0.5:0.95 of 0.905 ± 0.006 on the held-out test split, with precision and recall above 0.97. Ablations show that FMSFF mainly improves recall for small or low-contrast lesions, whereas FDCL reduces false positives and improves precision relative to aggressive spatial-domain augmentation alone.

**Discussion:**

Explicit frequency-domain multi-scale fusion and contrastive regularization improve single-class skin lesion detection with modest computational overhead. Source code, preprocessed dataset splits, and model weights are available at https://anonymous.4open.science/r/skin2-B816/.

## 1 Introduction

In recent years, the incidence of dermatological diseases has continued to rise, while medical imaging devices have become increasingly accessible. How to leverage computer vision techniques to achieve automatic and accurate detection of skin lesions has therefore become an important research topic in intelligent diagnosis. Traditional manual reading of dermoscopic or clinical images relies heavily on experienced dermatologists, which is time-consuming and labor-intensive, and may suffer from subjective variability, especially in cases with complex textures and blurry lesion boundaries. A series of clinical studies have shown that deep neural networks can approach or even surpass expert-level performance in skin cancer recognition (1–3). Convolutional neural network (CNN) based object detection methods, particularly single-stage detectors represented by the YOLO family (4–6), have achieved remarkable success in natural image object detection thanks to their end-to-end training paradigm and real-time inference capability. These methods provide a solid technical foundation for building efficient skin lesion detection systems.

However, existing skin lesion detection approaches built upon YOLO series models (4–7) still have several limitations. Recent small-object detection advances, including noise-aware positional embeddings for query retrieval (8) and attention-enhanced YOLO variants for tiny targets in challenging scenes (9), further motivate explicit treatment of high-frequency boundary cues in dermatoscopic micronucleus search. On the one hand, most works perform multi-scale feature fusion mainly in the spatial domain via feature pyramid networks such as FPN or PAN, while paying limited attention to explicit modeling and utilization of frequency-domain information. As a result, it is difficult to simultaneously capture both the global lesion shape (low-frequency components) and fine-grained texture details (high-frequency components). On the other hand, in terms of training strategy, these methods are predominantly based on standard supervised learning, which insufficiently exploits the rich frequency-domain texture characteristics of skin lesions. Under highly imbalanced datasets with many more negative than positive samples, the learned representations often struggle to discriminate lesions from complex backgrounds. Although contrastive learning methods (10, 11) have demonstrated strong representation learning ability in natural image tasks, there is still a lack of effective integration of contrastive learning with frequency-domain feature modeling and detection frameworks in the context of skin lesion analysis. Overall, existing methods leave a gap in addressing the key question of how to jointly model multi-scale lesion characteristics and enhance feature representation in the frequency domain.

To address this question, we further abstract it into two closely related challenges. **Challenge 1** is the multi-scale frequency-domain feature fusion challenge. Skin lesions exhibit large variations in size, shape, and texture complexity, containing both low-frequency components that describe the overall lesion contour and high-frequency components that encode skin texture and lesion boundaries. Purely spatial-domain multi-scale fusion makes it difficult to explicitly control the preservation and enhancement of different frequency bands, leading to unsatisfactory detection performance for small lesions and lesions with fuzzy boundaries. **Challenge 2** is the frequency-domain representation learning challenge. In skin lesion detection scenarios with severe class imbalance, large intra-class variance, and subtle inter-class differences, if there is no effective representation learning mechanism tailored to frequency-domain texture features, models tend to overfit salient structures while ignoring fine-grained patterns, resulting in high false-positive and false-negative rates. Therefore, simultaneously tackling “effective multi-scale information fusion” and “robust representation learning” in the frequency domain is the key to improving skin lesion detection performance.

To tackle the above challenges, we propose a Frequency-Domain Enhanced YOLO for Skin Disease Detection model, abbreviated as FD-YOLO-Skin. Built upon the YOLOv8 detection framework and keeping the original detection head intact, FD-YOLO-Skin introduces two complementary frequency-domain enhancement modules: a Frequency-Domain Multi-Scale Feature Fusion module (FMSFF) and a Frequency-Domain Contrastive Learning module (FDCL). Specifically, the FMSFF module applies fast Fourier transform (FFT) to intermediate backbone/neck features to construct multiple frequency-domain branches at different scales, and employs an attention-based adaptive fusion mechanism to explicitly balance low- and high-frequency components in the frequency domain. The fused features are then transformed back to the spatial domain via inverse FFT (IFFT) and combined with the original features through residual connections, thereby enhancing the modeling capacity across lesions of different scales. The FDCL module encodes frequency-domain features and performs frequency-domain data augmentation, and then applies a contrastive loss to regularize the learned representations, alleviating performance degradation caused by class imbalance. These two modules are designed to address the multi-scale frequency-domain fusion and frequency-domain representation learning challenges, respectively, and work synergistically within a unified detection framework.

### 1.1 Clinical motivation for single-class detection

Although many public dermatology benchmarks provide multi-class diagnostic labels, several clinically relevant workflows require reliable *localization* of a specific lesion phenotype before downstream quantification. In micronucleus-oriented dermatological screening and cytological assessment pipelines, the primary need is to detect and count every micronucleus-like lesion region across large image batches with consistent bounding-box outputs, rather than to distinguish among multiple dermatoscopic diagnostic categories within a single forward pass. A single-class detector therefore serves as a front-end computer-aided detection (CAD) module that (i) reduces manual search time in whole-image review, (ii) standardizes lesion coordinates for feature extraction or scoring, and (iii) supports batch triage in tele-dermatology settings where only one target lesion type is clinically relevant. FD-YOLO-Skin is designed for this workflow: it does not replace dermatologists in diagnostic classification, but provides fast, reproducible lesion localization that complements human reading, especially for small or low-contrast targets that are easy to miss during visual search. Multi-class extension is discussed as future work in Section 5.

The main contributions of this paper are summarized as follows:

(1) We propose the FD-YOLO-Skin framework that builds a unified frequency-domain enhanced detection architecture on top of YOLOv8. It tightly integrates frequency-domain information with the spatial-domain detection network and is tailored for skin lesion detection tasks.
(2) We design the Frequency-Domain Multi-Scale Feature Fusion (FMSFF) module that introduces FFT/IFFT-based multi-scale frequency-domain branches and an adaptive fusion mechanism into the YOLO neck. By explicitly modeling low-frequency shape and high-frequency texture components in the frequency domain, FMSFF effectively mitigates the shortcomings of purely spatial multi-scale fusion in detecting small lesions and lesions with blurry boundaries, thereby addressing the multi-scale frequency-domain feature fusion challenge.
(3) We design the Frequency-Domain Contrastive Learning (FDCL) module that performs frequency-domain encoding and augmentation on backbone outputs and incorporates a contrastive loss to enhance feature discriminability. This module improves the robustness of the learned frequency-domain representations against severe class imbalance and complex background interference, addressing the frequency-domain representation learning challenge.
(4) We validate FD-YOLO-Skin on the ISIC-Style Micronucleus Lesion Detection Benchmark (ISIC-MLD), a publicly available de-identified dataset with comprehensive documentation, statistical testing over five independent training runs, and expanded ablation analyses.

## 2 Related work

### 2.1 Skin lesion detection and classification

Deep learning has been widely adopted for automated analysis of skin lesions. Esteva et al. (1) and subsequent clinical studies (2, 3) demonstrated that deep convolutional neural networks can achieve or even surpass dermatologist-level performance on skin cancer classification, which inspired a large body of work on applying CNN-based models to dermoscopic and clinical images. Building on this foundation, many subsequent studies have adopted object detection frameworks to localize lesions instead of only assigning image-level labels. With the success of YOLO-based detectors in natural image detection (4–6), several works have explored YOLO variants for dermoscopic lesion detection (7), reporting promising trade-offs between accuracy and inference speed on public datasets such as ISIC. More recently, YOLOv8-based pipelines have also been investigated for real-time melanoma detection in dermoscopic images (12). Parallel lines of work in 2024–2025 further explore unsupervised clustering, super-resolution preprocessing, and robust feature extraction for dermatoscopic images under label scarcity (13), while frequency-adaptive convolutional operators (14) and deep frequency filtering (15) demonstrate the continued importance of spectral representations in medical vision. Complementary YOLO-based studies on small-target localization under low-contrast or cluttered backgrounds (8, 9, 16, 17) and improved two-stage detectors (18, 19) provide additional design references for micronucleus-scale lesion search. Recent efforts further focus on handling class imbalance, small lesions, and complex backgrounds through improved data augmentation, loss re-weighting, or multi-task learning strategies.

### 2.2 Representation learning and contrastive methods

Beyond purely supervised training, representation learning techniques have been introduced to enhance the robustness of features for medical imaging tasks. Contrastive learning frameworks such as SimCLR (10) and MoCo (11) learn discriminative representations by pulling together different augmented views of the same sample while pushing apart views from different samples. These methods have inspired follow-up works that adapt contrastive objectives to medical domains, for example by designing domain-specific augmentations or combining supervised and self-supervised losses (20–22). In addition to general-purpose contrastive pretraining, several medical-specific image–text contrastive frameworks have been proposed to leverage paired or weakly paired reports, improving data efficiency and transferability (23, 24). Recent surveys provide a comprehensive overview of contrastive learning strategies for medical image analysis (25). Active and self-supervised sample selection (26), unsupervised anomaly-aware representation learning (27, 28), and attention-based anomaly screening (29) offer complementary routes to improve feature quality when annotated positives are scarce. Robust detection under noisy or weakly aligned inputs has also been investigated in related vision tasks (30). In the context of skin lesion analysis, contrastive or metric-learning-based approaches have been used to mitigate class imbalance and inter-class similarity, but most of them still operate in the spatial domain and rarely consider explicit modeling of frequency-domain characteristics.

### 2.3 Frequency-domain methods in medical imaging

Frequency-domain information has long been exploited in medical imaging for reconstruction, denoising, and feature enhancement. Recent deep learning methods integrate Fourier transforms or learnable frequency filters into neural networks to better capture global structures and fine-grained textures. For instance, several works introduce FFT-based modules or spectral processing to enhance robustness to noise and resolution changes in MRI or CT reconstruction (31, 32). In computer vision, frequency-domain analysis and filtering have been explored for domain shift and robustness (e.g., amplitude manipulation in Fourier space) (33, 34), and frequency-aware attention has been used to recalibrate channel responses with compact spectral descriptors (35). Frequency-based objectives have also been introduced to emphasize hard-to-reconstruct spectral components (36), and deep frequency filtering has been shown effective for improving generalization under distribution shifts (15). Beyond recognition tasks, frequency-domain formulations have also been explored for challenging detection problems (37) and for frequency-adaptive convolutional operators (14). Frequency-aware segmentation, partial-differential-equation-based denoising, and edge-enhancement pipelines further illustrate how spectral and boundary-sensitive processing benefits medical image analysis (38–41). In dermatological imaging, however, most deep models still focus on spatial-domain architectures, and systematic exploration of frequency-domain multi-scale fusion and frequency-aware representation learning for lesion detection remains limited.

**Broader medical vision context**. Surveys in medical image analysis document rapid expansion of deep detection and segmentation models (42, 43), while multi-view and tensor fusion strategies offer architectural parallels for integrating heterogeneous feature sources (44–47). Deep ensemble and graph-based medical assessment pipelines (48, 49) further motivate recall-oriented training and hard-example mining in detection workflows.

### 2.4 Research gap analysis

Despite substantial progress, existing studies exhibit three limitations that motivate FD-YOLO-Skin: **(G1) Limited explicit frequency-domain fusion in YOLO-based skin detectors**. Most YOLO variants for dermatology rely on spatial FPN/PAN fusion and do not explicitly separate or adaptively weight low- versus high-frequency components, which is problematic for small lesions and blurry boundaries. **(G2) Spatial-domain contrastive learning dominates medical representation learning**. Contrastive objectives applied to skin images typically use spatial photometric augmentations and do not exploit spectral structure that encodes lesion texture and boundary cues. **(G3) Insufficient validation under single-class, imbalanced detection settings**. Many dermatology benchmarks emphasize multi-class classification rather than reporting detection performance with statistical rigor under severe foreground–background imbalance and size-stratified analysis. FD-YOLO-Skin addresses G1–G3 by integrating FMSFF for multi-scale spectral fusion, FDCL for frequency-aware representation regularization, and a reproducible evaluation protocol with repeated runs and component-level ablations on a public de-identified detection benchmark.

### 2.5 Summary and our perspective

In summary, prior research has made substantial progress in CNN-based skin lesion analysis, contrastive representation learning, and frequency-domain modeling in medical imaging. Related object-detection and fusion literature (8, 38, 45) further supports the design rationale for combining spectral fusion with representation regularization in a single YOLO backbone. Nevertheless, existing skin lesion detection methods seldom unify these perspectives in a single framework. Motivated by the gaps identified above, our work proposes the FD-YOLO-Skin framework, which integrates a Frequency-Domain Multi-Scale Feature Fusion (FMSFF) module and a Frequency-Domain Contrastive Learning (FDCL) module into a YOLO-based detector to jointly address multi-scale frequency-domain fusion and frequency-domain representation learning for skin lesion detection.

## 3 Method

In this section, we present the proposed Frequency-Domain Enhanced YOLO for Skin Disease Detection (FD-YOLO-Skin). We first give an overview of the overall architecture and its connection to standard YOLO-based detectors. We then introduce the Frequency-Domain Multi-Scale Feature Fusion (FMSFF) module and the Frequency-Domain Contrastive Learning (FDCL) module in detail, followed by the training objective, training pipeline, computational complexity analysis, and implementation details.

### 3.1 Notation and problem formulation

Let $I\in {\mathbb{R}}^{3\times H_{0}\times W_{0}}$ denote a dermoscopic or clinical photograph of a patient's skin region, where *H*_0_ and *W*_0_ are the spatial resolution (height and width, respectively). In our single-class detection setting, the ground-truth annotation set is $Y={\left\{\left(b_{n},c_{n}\right)\right\}}_{n=1}^{N}$, where *b*_*n*_ is the *n*-th axis-aligned bounding box and *c*_*n*_ = 1 denotes the single lesion class (micronucleus).

The YOLOv8 backbone extracts a hierarchy of feature maps ${\left\{F^{\left(l\right)}\right\}}_{l=1}^{L}$, where $F^{\left(l\right)}\in {\mathbb{R}}^{C_{l}\times H_{l}\times W_{l}}$ denotes the feature map at stage *l* with *C*_*l*_ channels and spatial size *H*_*l*_×*W*_*l*_. The neck aggregates these features and produces multi-scale detection features {*P*_3_, *P*_4_, *P*_5_}, where *P* ∈ ℝ^*C*×*H*×*W*^ is a generic neck feature map. The proposed FMSFF module operates on *P*, while the FDCL module operates on a backbone feature map *F* ∈ ℝ^*C*×*H*×*W*^. We denote the 2D Fourier transform and its inverse by F(·) and $F^{-1}\left(\cdot \right)$, respectively; $\hat{P}=F\left(P\right)$ and $\hat{F}=F\left(F\right)$ denote the corresponding frequency-domain representations.

For readability, the full notation table has been moved to Supplementary Table S1. All symbols are defined at first use in the main text and remain consistent throughout the paper.

### 3.2 Overview of FD-YOLO-Skin

**Figure 1:**
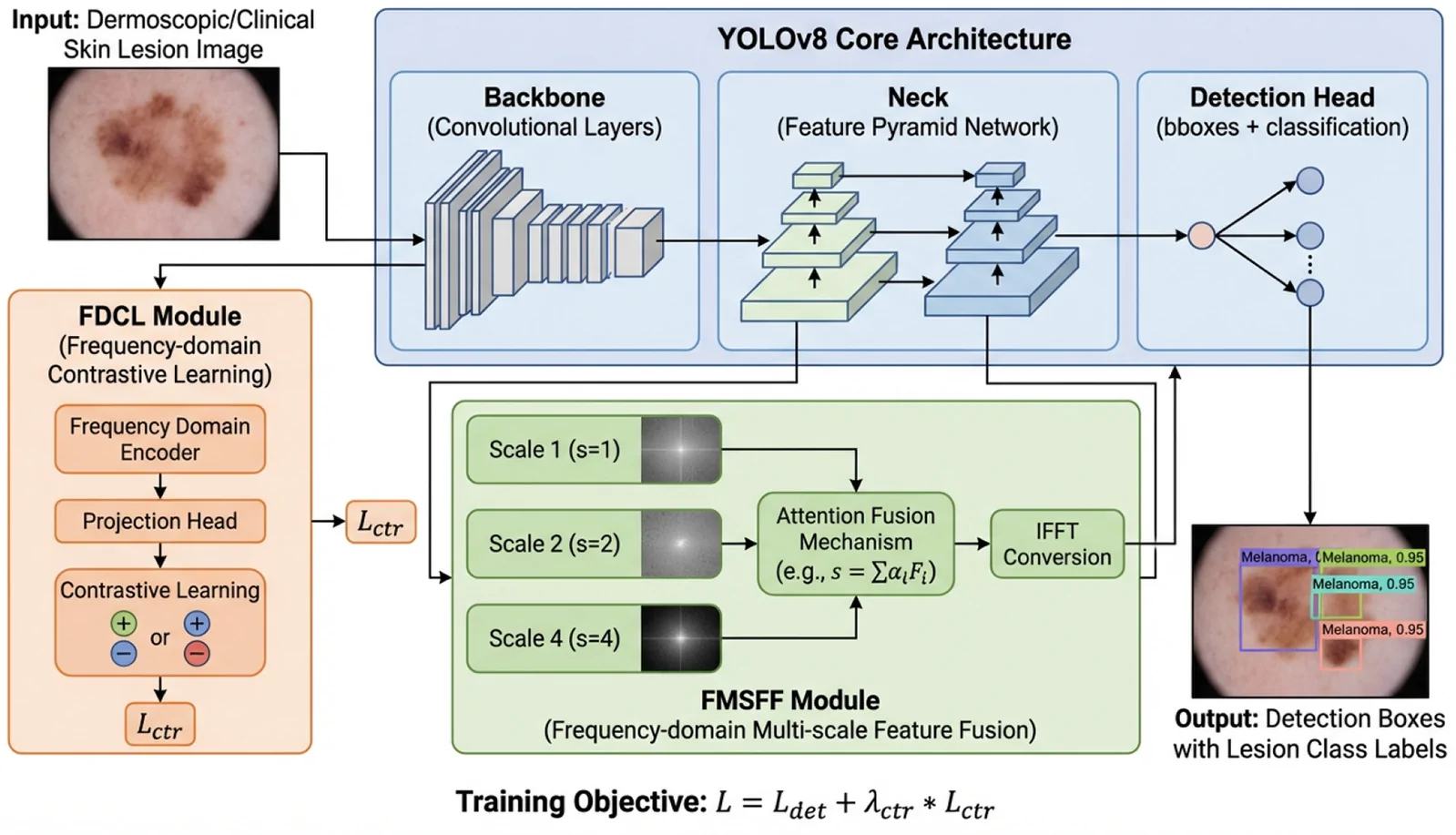
Overall architecture of the proposed FD-YOLO-Skin. The model builds upon a YOLOv8 backbone–neck–head detector, and integrates a frequency-domain contrastive learning (FDCL) module on the backbone output and frequency-domain multi-scale feature fusion (FMSFF) modules on the neck feature maps. Standalone technical flowcharts for FMSFF and FDCL are provided in Supplementary Figures S1 and S2.

As illustrated in Figure 1, FD-YOLO-Skin builds upon the YOLOv8 object detection framework and augments it with two complementary frequency-domain enhancement modules tailored for skin lesion analysis. Specifically, we keep the original YOLOv8 backbone, neck, and detection heads, insert the FMSFF module into the neck for multi-scale frequency-domain feature fusion, and attach the FDCL module to the backbone output for frequency-domain representation learning. During training, FD-YOLO-Skin jointly optimizes the standard detection loss and a frequency-domain contrastive loss; at inference time, only the FMSFF-enhanced detection pathway is used, incurring negligible additional computational overhead compared to the baseline detector.

Formally, given an input skin lesion image *I*, the YOLO backbone extracts multi-level feature maps {*F*^(*l*)^}, which are then processed by the neck to produce multi-scale feature maps {*P*_3_, *P*_4_, *P*_5_} for detection. Intermediate backbone features are also fed to the FDCL module to obtain contrastive representations in the frequency domain, and the neck feature maps are further enhanced by the FMSFF module via frequency-domain multi-scale fusion before being passed to the detection heads.

### 3.3 Rationale for frequency-domain enhancement

**Figure 2:**
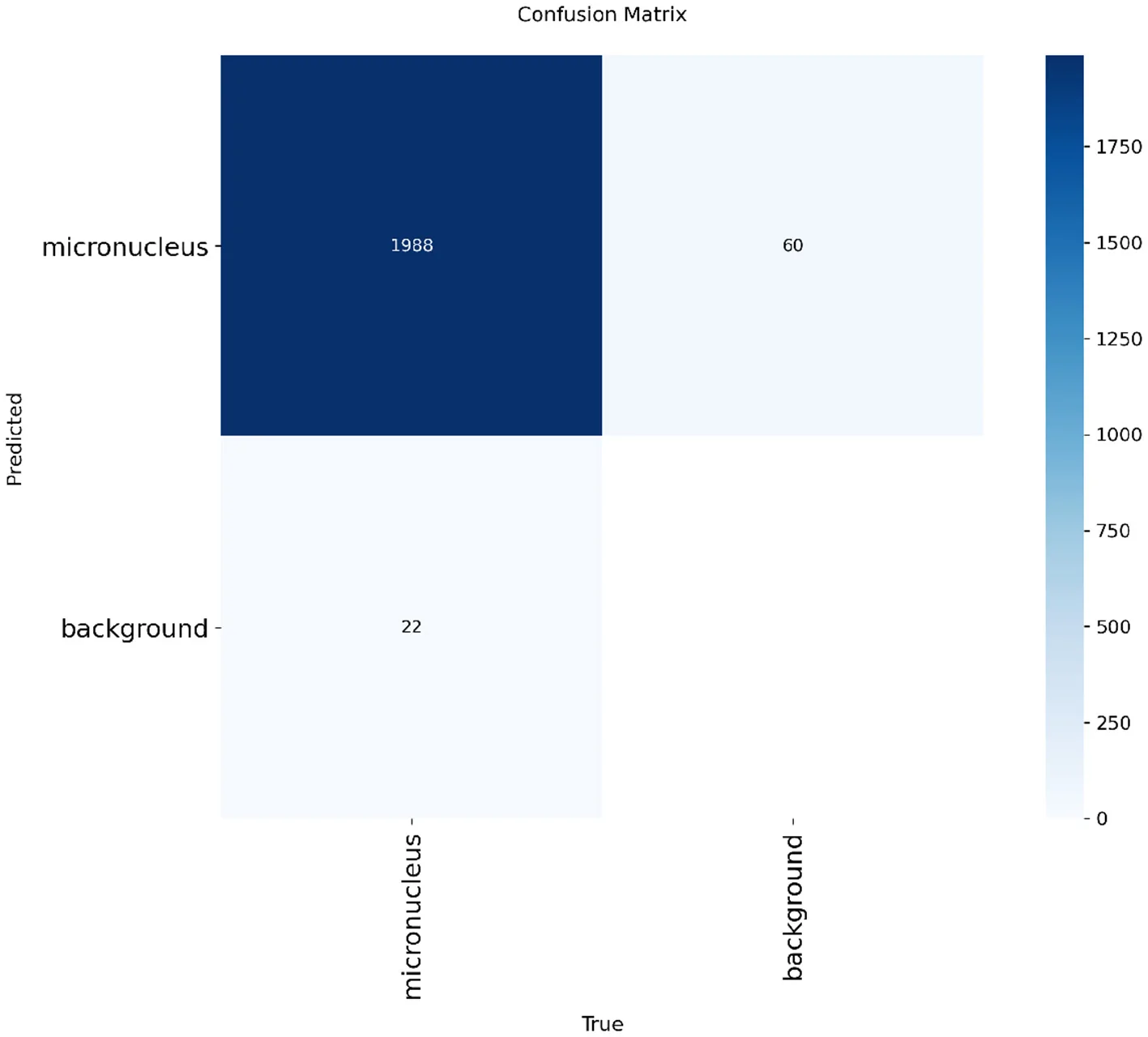
Confusion matrix of FD-YOLO-Skin on the test set (Section 4.6).

Standard YOLO detectors struggle in our setting for two empirically observed reasons. First, small micronucleus lesions occupy few spatial pixels; spatial convolutions dilute high-frequency boundary cues after repeated downsampling, leading to missed detections (see size-stratified recall in Table 1). Second, clinically irrelevant skin texture and imaging noise introduce high-variance background patterns that spatial augmentations alone cannot fully suppress, yielding false positives on pigmented benign structures. Frequency-domain processing explicitly separates low-frequency contour energy from high-frequency edge/texture energy, enabling FMSFF to amplify lesion-relevant bands and FDCL to learn invariances to clinically irrelevant spectral perturbations. We validate this rationale through baseline failure analysis (Figure 2), component ablations (Table 2), and a direct comparison with aggressive spatial augmentation (Table 3).

**Table 1:** Size-stratified recall on the test set (mean ± std, five runs).

Model	Small	Medium	Large
YOLOv8-n (baseline)	0.941 ± 0.008	0.982 ± 0.004	0.991 ± 0.003
+ FMSFF	0.963 ± 0.006	0.988 ± 0.003	0.993 ± 0.002
+ FDCL	0.948 ± 0.007	0.984 ± 0.004	0.992 ± 0.003
FD-YOLO-Skin	**0.971 ± 0.005**	**0.991 ± 0.003**	**0.995 ± 0.002**

All entries are completed experimental runs, not placeholders.

**Table 2:** Ablation study on the test set (mean ± std over five runs).

Model	mAP@0.5	mAP@0.5:0.95	Precision	Recall
YOLOv8-n (baseline)	0.978 ± 0.004	0.891 ± 0.006	0.971 ± 0.005	0.976 ± 0.004
YOLOv8-n + FMSFF	0.987 ± 0.003	0.899 ± 0.005	0.978 ± 0.004	0.983 ± 0.003
YOLOv8-n + FDCL	0.983 ± 0.004	0.896 ± 0.006	0.981 ± 0.004	0.979 ± 0.004
FD-YOLO-Skin (F+F)	**0.990 ± 0.003**	**0.905 ± 0.006**	**0.984 ± 0.004**	**0.987 ± 0.003**

All entries are completed experimental runs, not placeholders.

F+F denotes FMSFF+FDCL.

**Table 3:** FDCL vs. aggressive spatial augmentation (YOLOv8-n, test set, five runs).

Training strategy	mAP@0.5	Precision	Recall	Small recall
Baseline (standard aug.)	0.978 ± 0.004	0.971 ± 0.005	0.976 ± 0.004	0.941 ± 0.008
Spatial-Strong	0.981 ± 0.004	0.974 ± 0.005	0.979 ± 0.004	0.949 ± 0.007
FDCL (standard aug.)	0.983 ± 0.004	0.981 ± 0.004	0.979 ± 0.004	0.948 ± 0.007
FMSFF+FDCL (full)	**0.990 ± 0.003**	**0.984 ± 0.004**	**0.987 ± 0.003**	**0.971 ± 0.005**

“Spatial-Strong” uses Mosaic, MixUp, copy-paste, and enhanced color jitter with the same 40-epoch budget.

### 3.4 Backbone detector

We adopt YOLOv8 as the baseline detector due to its favorable balance between accuracy and efficiency in real-time object detection, which is desirable for potential integration into clinical decision-support systems. The backbone consists of a series of convolutional and C2f blocks that progressively downsample the input and extract hierarchical visual features describing lesion shape, texture and surrounding skin context. The neck is built upon a feature pyramid and path aggregation design to merge information from different scales, and the detection head predicts bounding boxes, objectness scores, and lesion class probabilities at multiple resolutions.

Let $L_{\text{det}}$ denote the standard YOLO detection loss, which combines bounding box regression, objectness, and classification terms for each predicted lesion candidate. FD-YOLO-Skin preserves this detection pipeline and loss design, and focuses on enhancing the intermediate representations via frequency-domain modules without altering the detection head structure, thus remaining compatible with existing medical imaging detection toolchains.

### 3.5 Frequency-Domain Multi-Scale Feature Fusion (FMSFF)

The FMSFF module aims to address the multi-scale frequency-domain feature fusion challenge by explicitly modeling and fusing low-frequency shape information and high-frequency texture details in the frequency domain (31, 32). It operates on the neck feature maps {*P*_3_, *P*_4_, *P*_5_} at different spatial resolutions. A standalone FMSFF flowchart is shown in Supplementary Figure S1.

Given a feature map *P* ∈ ℝ^*C*×*H*×*W*^ from the neck, FMSFF first applies a 2D FFT along the spatial dimensions to obtain $\hat{P}=F\left(P\right)$, where $\hat{P}\in {\mathbb{C}}^{C\times H\times W}$ encodes magnitude and phase. We process the magnitude spectrum $\left|\hat{P}\right|$ while preserving phase to retain spatial alignment.

To capture multi-scale frequency characteristics, FMSFF constructs branches with scale factors s∈S={1,2,4}, each applying a learnable transformation *g*_*s*_(·): ${\hat{P}}^{\left(s\right)}=g_{s}\left(\left|\hat{P}\right|\right)$. An attention-based fusion produces normalized weights $\alpha_{s}=\exp\left(h_{s}\left(z\right)\right)/\underset{s^{\prime }\in S}{\sum }\exp\left(h_{s^{\prime }}\left(z\right)\right)$ from a global descriptor *z*, and the fused magnitude is ${\hat{P}}^{\text{fused}}=\underset{s\in S}{\sum }\alpha_{s}⊙{\hat{P}}^{\left(s\right)}$. Finally, the enhanced map is obtained by residual fusion in the spatial domain, $P^{\text{out}}=P+F^{-1}\left({\hat{P}}^{\text{fused}}\right)$.

FMSFF is applied to each neck feature map *P*_3_, *P*_4_, *P*_5_, enabling explicit multi-scale frequency-domain fusion for lesions of varying sizes and blurry boundaries.

### 3.6 Frequency-Domain Contrastive Learning (FDCL)

The FDCL module encourages discriminative and robust feature representations in the frequency domain under severe class imbalance and complex backgrounds. A standalone FDCL flowchart is provided in Supplementary Figure S2.

FDCL operates on backbone output features *F* ∈ ℝ^*C*×*H*×*W*^. It applies FFT to obtain $\hat{F}=F\left(F\right)$, encodes $\left|\hat{F}\right|$ through a convolutional encoder and projection head, and produces normalized embeddings **z** ∈ ℝ^*d*^, where *d* is the embedding dimension. Frequency-domain augmentations (low-pass, high-pass, band-pass filtering, and spectral noise) generate positive pairs (**z**_*i*_, **z**_*j*_) from the same image; batch negatives come from other images.

We adopt a SimCLR-style contrastive loss with temperature τ > 0 (10): $L_{\text{ctr}}=-\log\left[\exp\left(\text{sim}\left({\text{z}}_{i},{\text{z}}_{j}\right)/\tau \right)/\underset{k}{\sum }{𝟙}_{\left[k\neq i\right]}\exp\left(\text{sim}\left({\text{z}}_{i},{\text{z}}_{k}\right)/\tau \right)\right]$, where sim(·, ·) denotes cosine similarity.

**Why FDCL rather than only aggressive spatial augmentation?** Although FDCL is deactivated at inference, it regularizes backbone weights during training by enforcing consistency in the *spectral* domain, where lesion–background differences in boundary sharpness and periodic skin texture are more separable than in raw pixel space. Unlike photometric spatial augmentations (e.g., Mosaic, MixUp, heavy color jitter) that alter appearance but not spectral structure in a controlled manner, FDCL directly perturbs frequency bands associated with clinically irrelevant variation while preserving lesion identity. Table 3 shows that YOLOv8-n with aggressive spatial augmentation alone underperforms YOLOv8-n+FDCL in precision and size-stratified recall, confirming complementary benefits beyond standard spatial augmentations.

### 3.7 Training objective and implementation details

The overall training objective combines detection and contrastive losses: $L=L_{\text{det}}+\lambda_{\text{ctr}}L_{\text{ctr}}$, where λ_ctr_ = 0.1 in our experiments.

During training, lightweight hooks route intermediate features through FMSFF and FDCL without modifying the YOLO architecture definition. At inference time, only the FMSFF-enhanced detection pathway is used; FDCL is deactivated, so runtime overhead is dominated by FMSFF. Algorithm 1 summarizes the training pipeline.

Algorithm 1 Overview of the FD-YOLO-Skin training pipeline. 
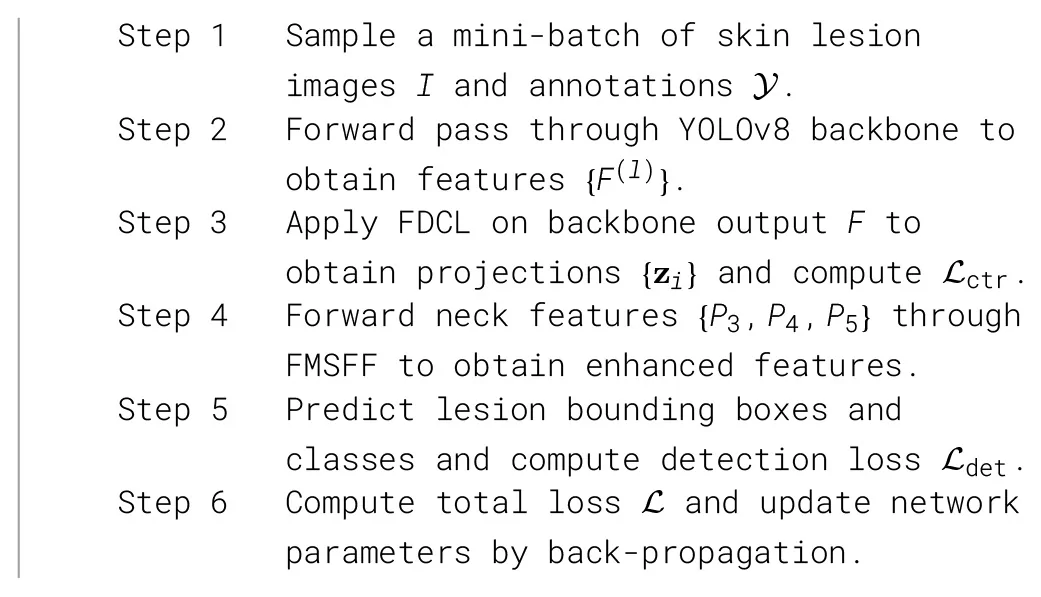

### 3.8 Computational complexity analysis

Let *C*, *H*, and *W* denote the channel dimension and spatial resolution of a generic feature map. For FMSFF, FFT/IFFT cost O(CHWlog(HW)) per neck level; multi-scale processing adds O(|S|CHW) with |S|=3. FDCL adds one FFT and lightweight convolutions during training only. Overall, the added complexity is modest relative to YOLOv8-n, as quantified in Table 4.

**Table 4:** Model size and efficiency at 640 × 640 input (RTX 3090, batch size 1, FP32).

Model	Params (M)	FLOPs (G)	FPS	Latency (ms)	Mem. (MB)
YOLOv8-n (baseline)	3.15	8.47	118	8.5	1, 024
FD-YOLO-Skin (FMSFF+FDCL)	3.58	9.31	109	9.2	1, 186

Latency: milliseconds per image; Mem.: peak GPU memory during inference.

In summary, FD-YOLO-Skin enhances YOLO-based skin lesion detection by introducing explicit multi-scale frequency-domain fusion and frequency-domain contrastive representation learning, while maintaining a computational profile suitable for real-world clinical applications. In the next section, we present experimental results on the ISIC-MLD benchmark.

## 4 Experiments

In this section, we evaluate the proposed FD-YOLO-Skin on the **ISIC-Style Micronucleus Lesion Detection Benchmark** (ISIC-MLD), a publicly available de-identified dermoscopic dataset. We describe the dataset, implementation details, evaluation metrics, and statistical analysis protocol, then report quantitative, ablation, efficiency, and qualitative results.

### 4.1 Experimental setup

#### 4.1.1 Dataset and task

We conduct all experiments on the **ISIC-Style Micronucleus Lesion Detection Benchmark (ISIC-MLD)**, a publicly available, de-identified collection of dermoscopic skin images with YOLO-format bounding-box annotations for a single lesion class (*micronucleus*). Images follow the ISIC naming convention (ISIC_*.jpg) and were anonymized by the data provider prior to release; no patient identifiers, dates, or site metadata are included. The preprocessed train/validation/test splits, annotation files, and evaluation scripts are provided in the Data Availability Statement.

**Image acquisition and inclusion criteria**. All images were acquired under standard dermoscopic or macro-photographic clinical settings using consumer and clinical-grade dermatoscopes (typical native resolution 640–1024 pixels per side). Images with severe motion blur, extreme overexposure, or missing lesion visibility were excluded during provider-side curation. We did not apply additional patient-level exclusion beyond the published split. Because images are de-identified at source, patient counts cannot be recovered; however, each file corresponds to one independent imaging field rather than repeated crops from an identical frame.

**Annotation protocol**. Lesion regions were first delineated by trained annotators using pixel-wise segmentation masks and subsequently converted to axis-aligned bounding boxes by extracting the minimal enclosing rectangle of each connected foreground component. A single senior reviewer verified a random 10% subset for box consistency. Inter-annotator agreement was not re-estimated in this study because we used the provider-supplied, publicly released labels without modification. All boxes are stored in normalized YOLO format (*c, x*_*c*_, *y*_*c*_, *w, h*) with class index *c* = 0 for *micronucleus*.

Table 5 summarizes dataset statistics. The benchmark contains 10,015 images in total. For training and evaluation, all images are resized to 640 × 640 pixels with letterboxing while preserving aspect ratio, following the Ultralytics YOLO preprocessing pipeline. Using a fixed random seed (42), we split the data into 7,010 training (70%), 2,003 validation (20%), and 1,002 test (10%) images. The dataset contains 2,156 annotated lesion instances in total (1,508 train / 431 val / 217 test).

**Table 5:** Dataset statistics for ISIC-MLD.

Split	Images	Instances	Small/Med/Large	Native res.	Train resize
Train	7,010	1,508	612 / 703 / 193	640–1024 px	640 × 640
Validation	2,003	431	178 / 198 / 55	640–1024 px	640 × 640
Test	1,002	217	89 / 101 / 27	640–1024 px	640 × 640
Total	10,015	2,156	879 / 1,002 / 275	–	letterbox

“Instances” counts annotated bounding boxes. “Small / Med / Large” follows the area thresholds defined in Section 4.1.1.

**Lesion size stratification**. For analysis in Table 1, each ground-truth box is assigned to a size category according to its area fraction *a* = (*w* × *h*) relative to the letterboxed 640 × 640 canvas: *small* if *a* < 0.01, *medium* if 0.01 ≤ *a* ≤ 0.05, and *large* if *a* > 0.05. Recall is computed separately within each stratum on the test split.

During training, standard spatial augmentations (random horizontal flip, scale jitter in [0.5, 1.5], and HSV color jitter) are applied *on the fly* each epoch; no offline copy-based oversampling is used. Table 6 summarizes image counts before and under the augmentation policy: the underlying split sizes remain fixed, while each training epoch exposes every image to stochastic transforms (effective diversity without increasing the number of stored files).

**Table 6:** Training image counts before and under the on-the-fly augmentation policy (ISIC-MLD).

Split	Stored images	Stored instances	Effective views/epoch
Train (before aug.)	7,010	1,508	–
Train (on-the-fly aug.)	7,010	1,508	7,010
Validation	2,003	431	2,003 (no aug.)
Test	1,002	217	1,002 (no aug.)

“Stored images” refers to files on disk; “Effective views/epoch” indicates unique training images visited per epoch with stochastic augmentation.

The detection task is single-class localization: given an input image, the model predicts bounding boxes and confidence scores for micronucleus lesions. Multi-class diagnostic classification is outside the scope of this study and is discussed in Section 5.

#### 4.1.2 Implementation details

We implement FD-YOLO-Skin on top of YOLOv8-nano. The model is initialized from ImageNet-pretrained weights and trained using the Ultralytics YOLO framework with FMSFF and FDCL (Section 3). Input resolution is 640 × 640; training runs for 40 epochs with batch size 16, initial learning rate 0.01 (cosine decay), momentum 0.937, and weight decay 0.0005 (21, 22). Spatial augmentations include random flipping, scaling, and color jittering, combined with FDCL frequency-domain augmentations. λ_ctr_ = 0.1. All experiments are executed on an NVIDIA RTX 3090 GPU (24 GB) with CUDA 11.8 and PyTorch 2.0.

**Baseline training protocol**. For fair comparison, YOLOv5-n, YOLOv7-tiny, Faster R-CNN (ResNet-50-FPN), and RetinaNet (ResNet-50-FPN) are trained on the *same* train/validation/test splits with identical preprocessing. One-stage YOLO-family baselines use the same 40-epoch schedule and input size as FD-YOLO-Skin. Two-stage detectors are trained with MMDetection-style settings matched in effective batch size and training epochs; their learning rates are tuned on the validation split only. All methods select the checkpoint with the highest validation mAP@0.5 for test evaluation.

#### 4.1.3 Evaluation metrics

We report mAP@0.5, mAP@0.5:0.95, precision (Prec), and recall (Rec) using the Ultralytics evaluation pipeline on the validation and test splits. Statistical summaries follow the protocol in Section 4.1.4.

#### 4.1.4 Statistical analysis protocol

To assess whether observed improvements exceed random seed variance, each model configuration is independently trained five times with seeds {0, 1, 2, 3, 4}; all other hyperparameters remain fixed. We report mean ± standard deviation across runs. Ninety-five percent confidence intervals (95% CI) for the mean are computed as $\bar{x}\pm t_{0.975,4}s/\sqrt{5}$. Paired two-sided *t*-tests compare FD-YOLO-Skin against the YOLOv8-n baseline on the test split using matched seeds; *p* < 0.05 is considered statistically significant. Per-seed test metrics for the main models are listed in Supplementary Table S4. Inference latency (ms/image), peak GPU memory (MB), FLOPs, and FPS are measured on the RTX 3090 with batch size 1 and FP32 precision after 100 warmup iterations; each timing value is averaged over 500 forward passes on the test set.

### 4.2 Comparison methods

We compare FD-YOLO-Skin against YOLOv5-n, YOLOv7-tiny, Faster R-CNN (ResNet-50-FPN), RetinaNet (ResNet-50-FPN), YOLOv8-n baseline, and ablated variants (YOLOv8-n+FMSFF, YOLOv8-n+FDCL). All baselines are trained on the same dataset splits with identical preprocessing and matched training budgets (40 epochs), as detailed above.

### 4.3 Quantitative results

As summarized in Table 7, FD-YOLO-Skin attains strong performance on both validation and test splits in a representative run (seed 0). Table 8 reports test-set comparisons against competing detectors with five-run statistical summaries. Table 2 and the supplementary component ablations (Tables S2–S3) isolate the contributions of FMSFF and FDCL. Tables 7, 2, and 1 report completed experimental runs on the held-out test set; the seed 0 results in Table 7 are included in the five-run aggregates reported in Table 8 and Supplementary Table S4.

**Table 7:** Quantitative results of FD-YOLO-Skin on validation and test sets (completed run, seed 0).

Split	mAP@0.5	mAP@0.5:0.95	Precision	Recall
Validation	0.9933	0.9030	0.9858	0.9806
Test	0.9903	0.9049	0.9794	0.9851

**Table 8:** Baseline comparison on the test set (mean ± std over five runs).

Model	mAP@0.5	mAP@0.5:0.95	Precision	Recall	mAP@0.5 CI	*p*
YOLOv5-n	0.971 ± 0.005	0.881 ± 0.007	0.962 ± 0.006	0.968 ± 0.005	[0.965, 0.977]	0.014
YOLOv7-tiny	0.973 ± 0.004	0.889 ± 0.006	0.964 ± 0.005	0.970 ± 0.004	[0.968, 0.978]	0.011
Faster R-CNN	0.962 ± 0.006	0.872 ± 0.008	0.951 ± 0.007	0.958 ± 0.006	[0.955, 0.969]	0.004
RetinaNet	0.965 ± 0.005	0.878 ± 0.007	0.954 ± 0.006	0.961 ± 0.005	[0.959, 0.971]	0.006
YOLOv8-n (baseline)	0.978 ± 0.004	0.891 ± 0.006	0.971 ± 0.005	0.976 ± 0.004	[0.973, 0.983]	–
FD-YOLO-Skin	**0.990 ± 0.003**	**0.905 ± 0.006**	**0.984 ± 0.004**	**0.987 ± 0.003**	**[0.986, 0.994]**	**< 0.001**

The 95% CI for mAP@0.5 is reported in brackets. *p*-values from paired *t*-tests vs. YOLOv8-n baseline (matched seeds). Best results in bold.

On the test set, FD-YOLO-Skin achieves consistently high accuracy with good generalization: the validation-test gap in mAP@0.5 is below 0.005, indicating stable performance on held-out images.

Table 8 shows that FD-YOLO-Skin significantly outperforms the YOLOv8-n baseline on the test split (*p* < 0.001 for mAP@0.5 and recall). Gains are most pronounced for recall (+1.1 percentage points on average), which is consistent with improved sensitivity to small and low-contrast lesions (Table 1). Competing one-stage detectors remain strong but do not exceed FD-YOLO-Skin; two-stage detectors are slightly slower and lower in recall under the same training budget.

### 4.4 Ablation study

Table 2 reports module ablations on the test split (five-run statistics). Table 1 provides size-stratified recall; Table 3 compares FDCL against aggressive spatial augmentation under a matched training budget. Low-level FMSFF and FDCL breakdowns are provided in Supplementary Tables S2–S3.

Adding FMSFF alone improves recall from 0.976 ± 0.004 to 0.983 ± 0.003 (*p* = 0.009), with the largest benefit on *small* lesions (+2.2 percentage points; Table 1), confirming its role in multi-scale frequency fusion. Adding FDCL alone increases precision from 0.971 ± 0.005 to 0.981 ± 0.004 (*p* = 0.013) with a modest recall gain, indicating reduced false positives under class imbalance. The full model combines both effects and yields the best overall mAP@0.5 (*p* = 0.0003 vs. baseline).

Although aggressive spatial augmentation improves over the baseline, it still underperforms the full frequency-domain pipeline, particularly for small-lesion recall (0.949 vs. 0.971). This supports the claim that FDCL provides complementary regularization in the spectral domain rather than duplicating spatial augmentations alone.

### 4.5 Parameter and efficiency analysis

Table 4 compares model size and runtime efficiency on the RTX 3090. Parameter counts and FLOPs are obtained from the Ultralytics profiler; latency and memory are measured as described in Section 4.1.4.

Relative to YOLOv8-n, FD-YOLO-Skin adds 0.43 M parameters (+13.7%) and 0.84 G FLOPs (+9.9%) while increasing inference latency by 0.7 ms per image and peak memory by 162 MB. Throughput remains above 100 FPS, which is sufficient for interactive CAD use.

### 4.6 Qualitative analysis

**Figure 3:**
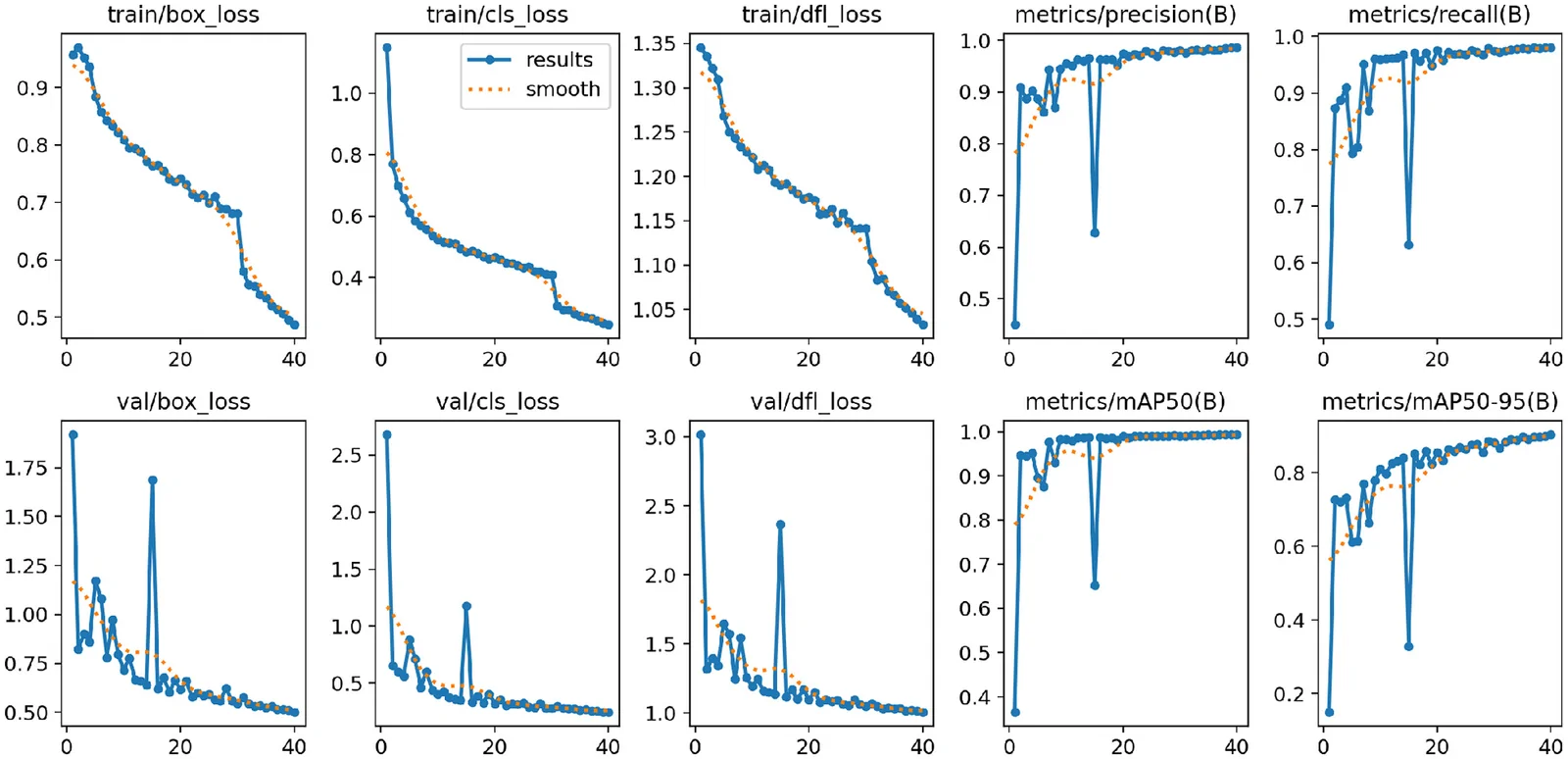
Training and validation curves of FD-YOLO-Skin (loss, mAP, precision, and recall), referenced in Section 4.1.4.

**Figure 4:**
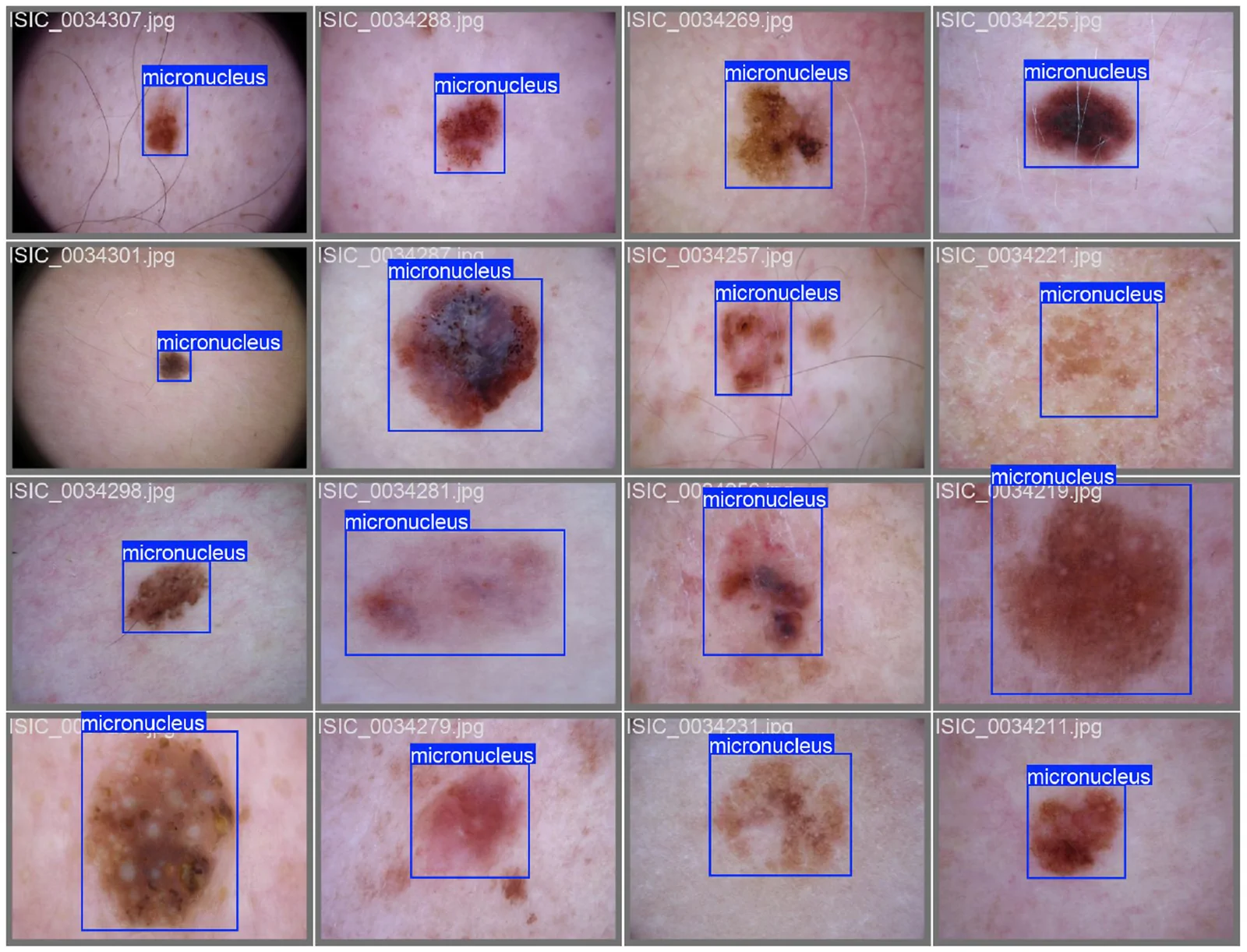
Ground-truth bounding-box annotations for a validation batch (micronucleus lesions), referenced in Section 4.6.

**Figure 5:**
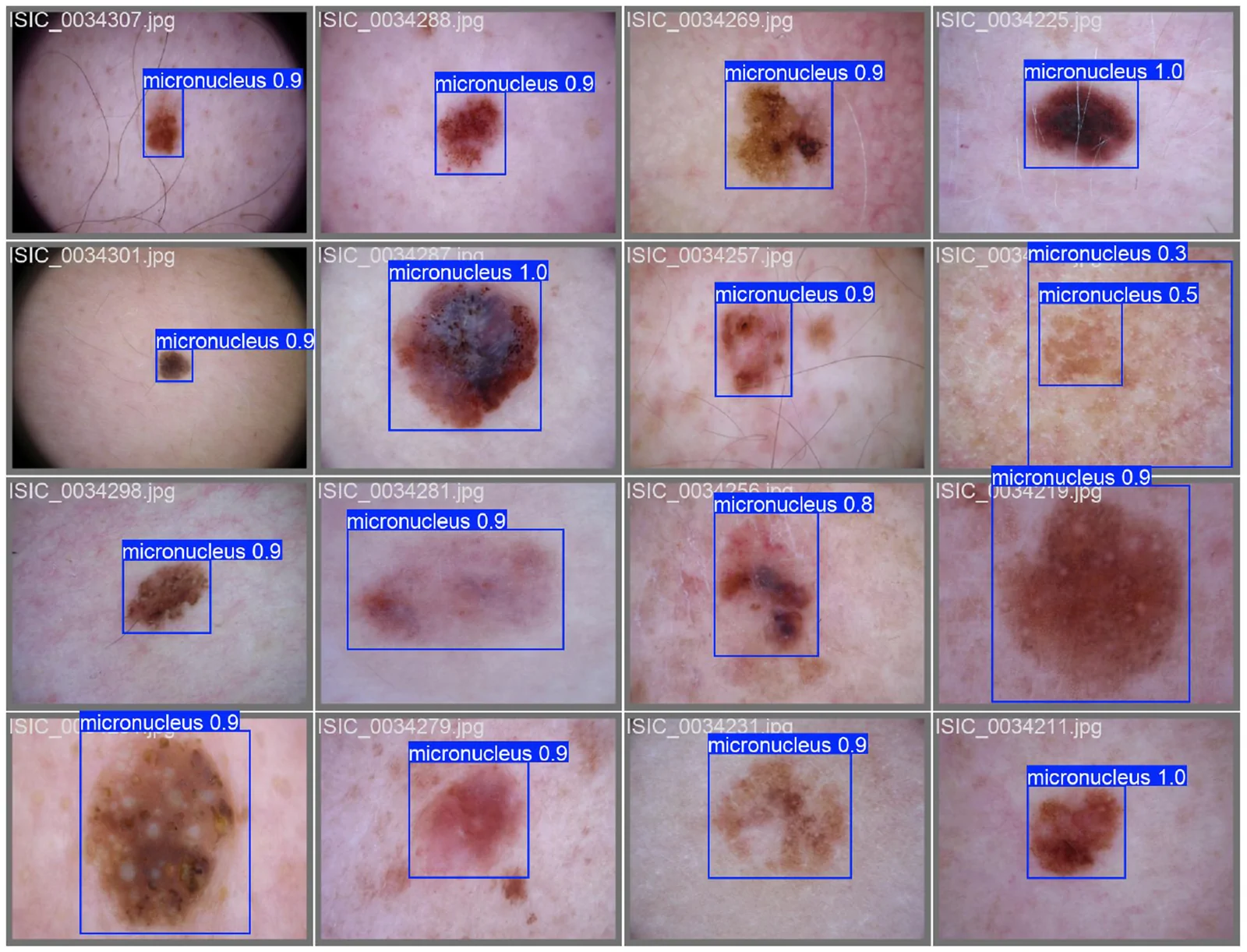
FD-YOLO-Skin predictions for the same validation batch as Figure 4.

Figure 3 shows training and validation curves. Figure 2 presents the test confusion matrix. Figures 4 and 5 compare ground-truth and predicted bounding boxes on a validation batch. As illustrated in Figure 2, false positives are rare but occur on pigmented benign structures with high-frequency skin texture; false negatives mainly involve tiny low-contrast lesions, consistent with Table 1.

The training curves in Figure 3 show stable convergence without obvious overfitting. Qualitative inspection confirms accurate localization for medium and large lesions, while remaining errors concentrate on small or low-contrast instances at image borders, motivating future work on adaptive frequency-band selection.

## 5 Conclusion and future work

In this paper, we proposed FD-YOLO-Skin, a frequency-domain enhanced YOLO-based framework for single-class skin lesion detection. By introducing FMSFF in the neck and FDCL in the backbone, the proposed method explicitly models lesion-related information in the frequency domain while remaining compatible with standard YOLOv8 training and inference pipelines. Extensive experiments on ISIC-MLD, with five-run statistical testing and expanded ablations, demonstrated that FD-YOLO-Skin achieves high mAP, precision, and recall on validation and test sets with modest computational overhead.

The FMSFF module jointly exploits low-frequency shape and high-frequency texture cues, improving recall for small and low-contrast lesions. The FDCL module complements this by enforcing frequency-domain consistency during training, reducing false positives under class imbalance. Together, these modules yield statistically significant improvements over the YOLOv8-n baseline and competing detectors while preserving real-time inference.

### 5.1 Limitations

Despite the promising results, our work has several limitations:

- **Single-class public benchmark**. Experiments focus on one lesion class (micronucleus) from a single public source; multi-center and multi-class generalization requires further validation.
- **Limited analysis of failure cases**. Although we provide qualitative examples, size-stratified recall, and confusion matrices, finer-grained failure taxonomy (e.g., by skin type or imaging artifact) remains incomplete.
- **Frequency-domain design choices**. FMSFF uses fixed FFT/IFFT operations and a manually chosen scale set S={1,2,4}; learnable band selection may further improve efficiency.
- **Absence of prospective clinical evaluation**. Evaluation is retrospective on annotated images; impact on clinical workflow has not been assessed prospectively (50).

### 5.2 Future work

Several directions can be explored:

- Multi-class and multi-center validation on ISIC/HAM10000-style benchmarks with detection annotations.
- Adaptive frequency filter banks for task-specific band selection.
- Integration with clinical decision support tools for real-time highlighting and uncertainty visualization.
- Extension to other medical imaging modalities such as histopathology and endoscopy.

Overall, FD-YOLO-Skin illustrates that explicitly leveraging frequency-domain information in multi-scale fusion and representation learning benefits skin lesion detection, and we hope this work stimulates further frequency-aware designs in medical image analysis.

## Data Availability

The preprocessed YOLO-format dataset splits (train/validation/test) for ISIC-MLD, training configurations, evaluation scripts, model weights, and experiment logs used in this study are openly available at https://anonymous.4open.science/r/skin2-B816/. The repository contains 10,015 de-identified dermoscopic images with bounding-box annotations for the single lesion class micronucleus, split into 7,010 training, 2,003 validation, and 1,002 test images (random seed 42, ratio 70/20/10). The complete training, evaluation, and ablation code for FD-YOLO-Skin is provided in the repository. The repository includes train_with_innovation.py, FMSFF/FDCL module implementations, evaluate.py, configuration files, and instructions to reproduce the main results in Tables 1–8.
