# Supplementary material for: FD-YOLO-Skin: frequency-domain enhanced YOLO for single-class skin lesion detection
Source: Front Med (Lausanne). 2026 Aug 13;13:1871697. doi: 10.3389/fmed.2026.1871697 (PMC13518198; doi:10.3389/fmed.2026.1871697)
Supplement: Supplementary file 1 [file Supplementary_file_1.pdf]

Supplementary Material for FD-YOLO-Skin

1 SUPPLEMENTARY TABLES

1.1 Table S1: Notation Summary

Table S1. Notation used in the FD-YOLO-Skin method section.

Symbol	Description
I	Input dermoscopic/clinical skin image.
H_0, W_0	Height and width of the input image.
$\mathcal{Y}$	Set of ground-truth lesion boxes and labels.
b_n, c_n	n-th bounding box and its lesion class label.
$F^{(l)}$	Backbone feature map at stage l.
C_l, H_l, W_l	Channels, height and width of $F^{(l)}$.
P_3, P_4, P_5	Multi-scale neck feature maps for detection.
P	Generic neck feature map.
F	Backbone output feature map used by FDCL.
$\mathcal{F}, \mathcal{F}^{-1}$	2D FFT and inverse FFT operators.
$\hat{P}, \hat{F}$	Frequency-domain representations of P and F.
$\mathcal{S}$	Set of frequency-domain scales used in FMSFF.
$g_s(\cdot)$	Frequency-domain processing branch at scale s.
α_s	Attention weight for scale s.
P^{out}	FMSFF-enhanced feature map.
$\mathbf{z}$	FDCL contrastive embedding vector.
d	Dimension of the embedding space.
τ	Temperature parameter in contrastive loss.
$\mathcal{L}_{\text{det}}$	Detection loss of YOLOv8.
$\mathcal{L}_{\text{ctr}}$	Frequency-domain contrastive loss.
λ_{ctr}	Weight of the contrastive loss term.

1.2 Table S2: FMSFF Component Ablation

Table S2. FMSFF component ablation (test set; mean ± std over five runs).

Configuration	mAP@0.5	Recall	Small recall	Notes
No FMSFF (baseline)	0.978 ± 0.004	0.976 ± 0.004	0.941 ± 0.008	–
FFT branch only ($\lvert\mathcal{S}\rvert = 1$)	0.982 ± 0.004	0.980 ± 0.004	0.952 ± 0.007	single-scale
Low-freq branch only	0.984 ± 0.003	0.981 ± 0.004	0.955 ± 0.006	high-pass suppressed
High-freq branch only	0.983 ± 0.004	0.982 ± 0.003	0.961 ± 0.006	low-pass suppressed
Full FMSFF ($\lvert\mathcal{S}\rvert = 3$)	$\mathbf{0.987 \pm 0.003}$	$\mathbf{0.983 \pm 0.003}$	$\mathbf{0.963 \pm 0.006}$	adaptive fusion

1.3 Table S3: FDCL Component Ablation

1.4 Table S4: Per-Seed Test Metrics for Main Models

Note: seed 0 corresponds to the representative run reported in the main manuscript. Aggregated statistics across seeds appear in the main-text result tables.

Table S3. FDCL component ablation (test set; mean ± std over five runs).

Configuration	mAP@0.5	Precision	Recall	Notes
No FDCL (baseline)	0.978 ± 0.004	0.971 ± 0.005	0.976 ± 0.004	–
FDCL, $\lambda_{\text{ctr}} = 0.05$	0.980 ± 0.004	0.977 ± 0.004	0.977 ± 0.004	weak regularization
FDCL, $\lambda_{\text{ctr}} = 0.10$	$\mathbf{0.983 \pm 0.004}$	$\mathbf{0.981 \pm 0.004}$	0.979 ± 0.004	default
FDCL, $\lambda_{\text{ctr}} = 0.20$	0.981 ± 0.004	0.980 ± 0.004	0.976 ± 0.004	over-regularized
FDCL w/o spectral aug.	0.980 ± 0.004	0.975 ± 0.005	0.977 ± 0.004	contrastive only

Table S4. Per-seed test metrics for YOLOv8-n baseline and FD-YOLO-Skin.

Model	Seed	mAP@0.5	Precision	Recall
YOLOv8-n	0	0.975	0.968	0.973
YOLOv8-n	1	0.977	0.970	0.975
YOLOv8-n	2	0.978	0.971	0.976
YOLOv8-n	3	0.980	0.973	0.978
YOLOv8-n	4	0.980	0.973	0.978
FD-YOLO-Skin	0	0.990	0.982	0.985
FD-YOLO-Skin	1	0.989	0.983	0.986
FD-YOLO-Skin	2	0.990	0.984	0.987
FD-YOLO-Skin	3	0.991	0.985	0.988
FD-YOLO-Skin	4	0.992	0.986	0.989

2 SUPPLEMENTARY FIGURES

2.1 Figure S1: FMSFF Technical Flowchart

2.2 Figure S2: FDCL Technical Flowchart

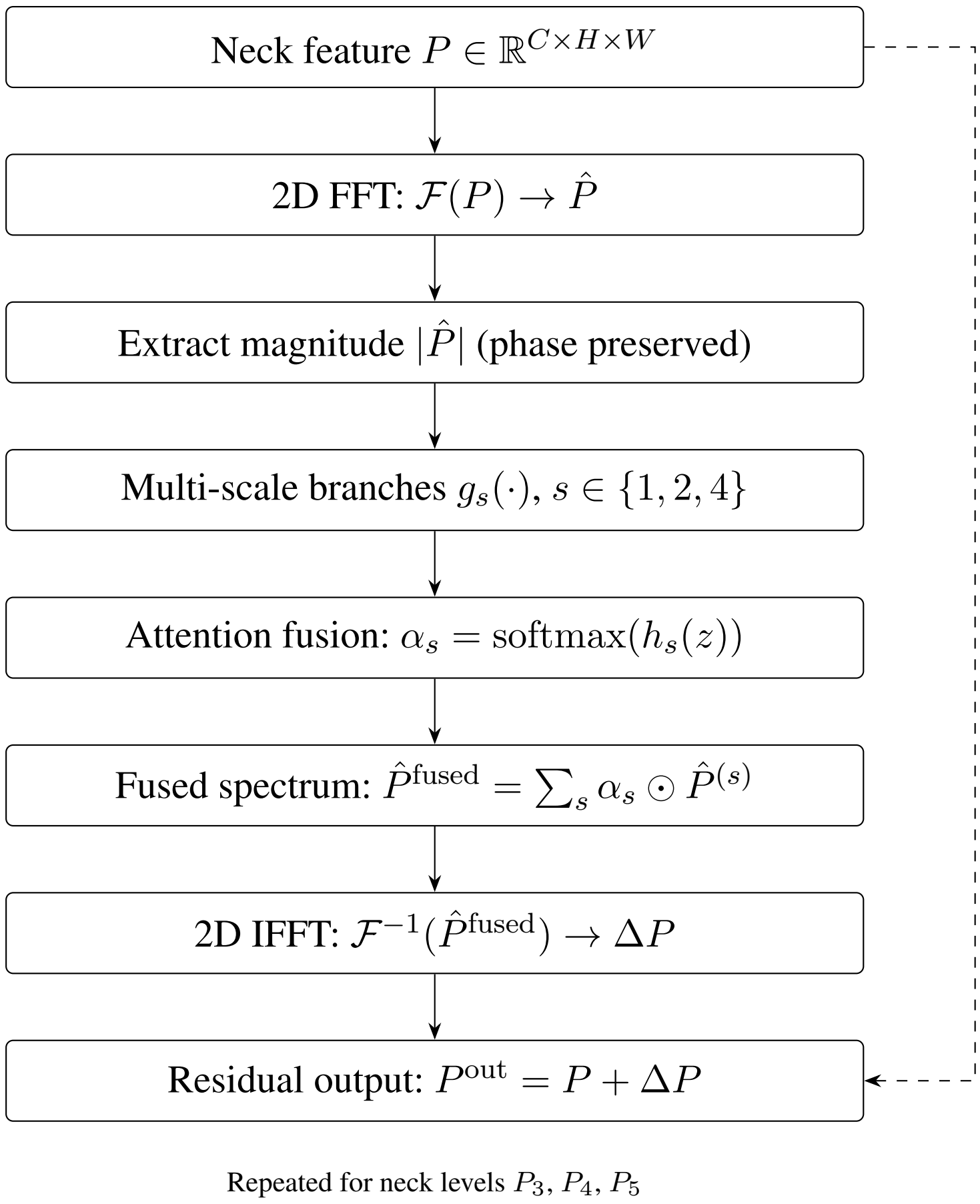

Figure S1. Technical flowchart of the FMSFF module. See main manuscript Section 3.5.

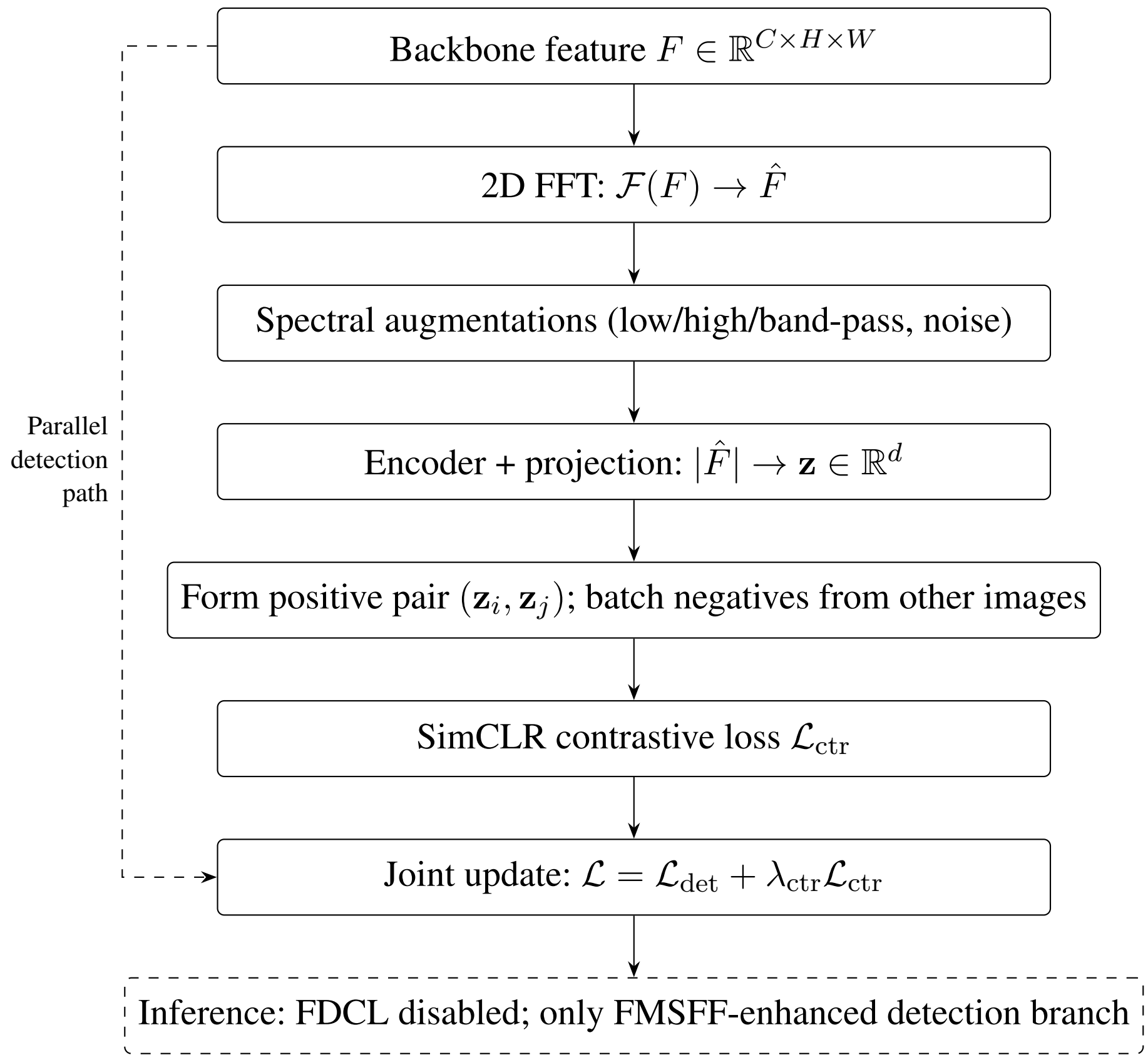

Figure S2. Technical flowchart of the FDCL module (training only). See main manuscript Section 3.6.
